# Virus-Bacteria Rice Co-Infection in Africa: Field Estimation, Reciprocal Effects, Molecular Mechanisms, and Evolutionary Implications

**DOI:** 10.3389/fpls.2017.00645

**Published:** 2017-05-01

**Authors:** Charlotte Tollenaere, Severine Lacombe, Issa Wonni, Mariam Barro, Cyrielle Ndougonna, Fatoumata Gnacko, Drissa Sérémé, Jonathan M. Jacobs, Eugénie Hebrard, Sebastien Cunnac, Christophe Brugidou

**Affiliations:** ^1^Interactions Plantes-Microorganismes-Environnement, Institut de Recherche pour le Développement (IRD), Cirad, Univ MontpellierMontpellier, France; ^2^Laboratoire Mixte International Patho-Bios, Laboratoire de Bactériologie, Institut de l'Environnement et de Recherches Agricoles (INERA)Bobo-Dioulasso, Burkina Faso; ^3^Laboratoire Mixte International Patho-Bios, Laboratoire de Virologie et de Biotechnologies Végétales, Institut de l'Environnement et de Recherches Agricoles (INERA)Kamboinsé, Burkina Faso

**Keywords:** co-infection, pathogen evolution, genotype-by-genotype effects, phytopathology, rice diseases, silencing mechanisms

## Abstract

Simultaneous infection of a single plant by various pathogen species is increasingly recognized as an important modulator of host resistance and a driver of pathogen evolution. Because plants in agro-ecosystems are the target of a multitude of pathogenic microbes, co-infection could be frequent, and consequently important to consider. This is particularly true for rapidly intensifying crops, such as rice in Africa. This study investigated potential interactions between pathogens causing two of the major rice diseases in Africa: the *Rice yellow mottle virus* (RYMV) and the bacterium *Xanthomonas oryzae* pathovar *oryzicola* (*Xoc*) in order to: 1/ document virus-bacteria co-infection in rice in the field, 2/ explore experimentally their consequences in terms of symptom development and pathogen multiplication, 3/ test the hypothesis of underlying molecular mechanisms of interactions and 4/ explore potential evolutionary consequences. Field surveys in Burkina Faso revealed that a significant proportion of rice fields were simultaneously affected by the two diseases. Co-infection leads to an increase in bacterial specific symptoms, while a decrease in viral load is observed compared to the mono-infected mock. The lack of effect found when using a bacterial mutant for an effector specifically inducing expression of a small RNA regulatory protein, HEN1, as well as a viral genotype-specific effect, both suggest a role for gene silencing mechanisms mediating the within-plant interaction between RYMV and *Xoc*. Potential implications for pathogen evolution could not be inferred because genotype-specific effects were found only for pathogens originating from different countries, and consequently not meeting in the agrosystem. We argue that pathogen-pathogen-host interactions certainly deserve more attention, both from a theoretical and applied point of view.

## Introduction

### Multiple infections in plant pathosystems

Plant pathologists have mostly focused on a tight pair of one plant-one pathogen interactions. However, there is accumulating evidence that various pathogen species or genotypes may co-exist within a single plant in agro-ecosystems (Barrett et al., 2009; Lamichhane and Venturi, 2015; Tollenaere et al., 2016), a phenomenon hereafter defined as co-infection or multiple infections. Investigations specifically designed to simultaneously document the incidence of various pathogen species remain few, but revealed high levels of co-infection (Malpica et al., 2006; Pagan et al., 2010).

During co-infection, the presence of co-infecting pathogens sharing the same host plant may affect the outcome of infection, both in terms of intra-host pathogen accumulation and symptom development (Tollenaere et al., 2016), as primarily reported for plant viruses (see for example Gil-Salas et al., 2012, and for a comprehensive review Syller, 2012), but was also shown across different kingdoms (see for example Le May et al., 2009; Shapiro et al., 2013; Orton and Brown, 2016) when investigated. Such pathogen-pathogen interactions may be direct or indirect and unpredictably synergistic or antagonistic (Tollenaere et al., 2016). These effects evidenced at individual plant levels may translate to the population level, with epidemiological (Zhang et al., 2001) and evolutionary (Alizon et al., 2013) consequences.

### Potential impact of genetic diversity on co-infection outcome

Co-infection may modify the selection pressure applied to each pathogen, and consequently have drastic consequences on the evolution of virulence (Alizon et al., 2013; Tollenaere et al., 2016). This would particularly be the case if the infection outcome is driven by the particular pathogen genotypes involved in co-infection (Bashey, 2015), but very few studies have tackled this issue experimentally. In trematode fish parasites, Seppala et al. (2009, 2012) demonstrated that the specific combination of pathogen genotypes (G_P_^*^G_P_ interactions) would determine infection success of each pathogen genotype in the context of co-infection. The favored genotype for each pathogen species depends on the presence/absence and even the genetic composition of the other parasite species. Similar to host genotype by parasite genotype interactions, that are a fundamental requirement for coevolution (Thompson, 2005), G_P_^*^G_P_ interactions may also have drastic consequences for evolutionary trajectories of co-infecting parasites, and could help in maintaining genetic variability within each pathogen species (Seppala et al., 2009, 2012; Bashey, 2015).

### Study system: virus-bacteria interactions in rice

**Figure 1:**
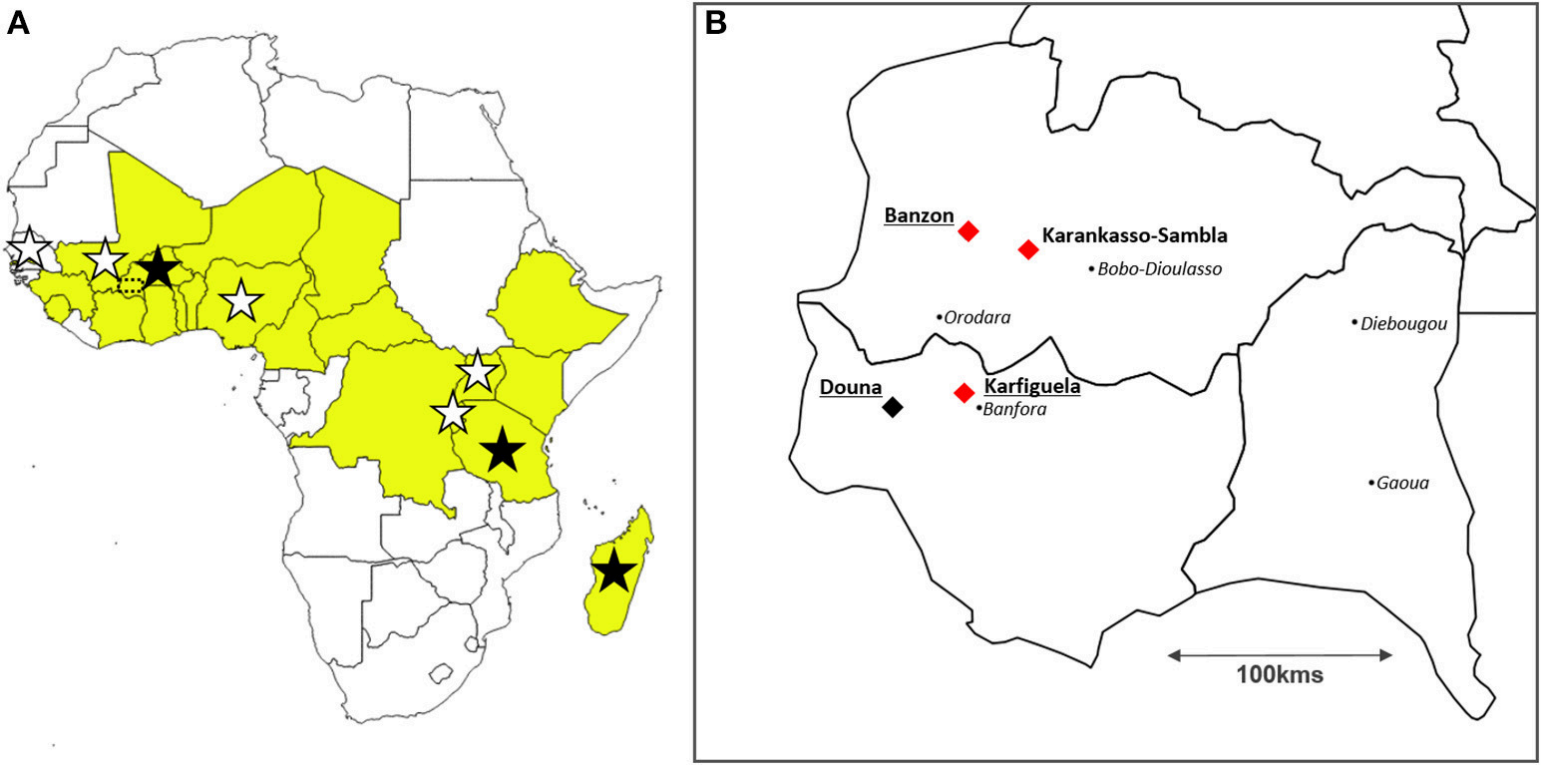
**Location of the study sites. (A)** Map of Africa displaying the countries where the bacterium *Xanthomonas oryzae* pv. *oryzicola* (stars) and the *Rice yellow mottle virus* (in yellow) have been reported to date; and location of the three countries from which strains used for experiments using pathogen genotypes sampled at the continental scale originate (dark stars): Burkina Faso, Tanzania, and Madagascar. Southwestern Burkina Faso (see **B**) is indicated with a dotted rectangle. **(B)** Location of the study sites in Burkina Faso represented with diamonds: the three sites (Banzon, Karankasso-Sambla and Karfiguela) where bacterial strains and viral isolates have been collected in 2014 to be used for experimental infections (local scale) are indicated with red diamonds, while the three irrigated rice perimeters where virus-bacteria co-occurence levels have been estimated in 2015 (Banzon, Karfiguela, and Douna) are underlined. The black points represent the main towns.

Rice (*Oryza* spp.) cultivation is increasing dramatically in Africa to face the rapidly growing demand (Wopereis et al., 2013). However, biotic constraints (such as diseases) impact negatively on rice production. In Africa, major rice pathogens include the *Rice yellow mottle virus* (RYMV) and bacteria of the *Xanthomonas oryzae* species (Séré et al., 2013). Both RYMV and *X. oryzae* have been reported in most African rice-growing regions and consequently share the same geographical range in Africa. They are both preferentially found in irrigated fields (Séré et al., 2013), where they represent a threat for the required intensification of rice cultivation in Africa. RYMV is a highly damaging sobemovirus restricted to Africa (Abo et al., 1998). It has been reported in most rice producing countries (Figure 1A). The gammaproteobacterium *X. oryzae* comprises two pathovars, both capable of infecting rice, but causing different diseases: *X*. *oryzae* pv. *oryzae* (*Xoo*) is responsible for Bacterial Leaf Blight (BLB) symptoms while *X. oryzae* pv. *oryzicola* (*Xoc*) induces Bacterial Leaf Streak (BLS) symptoms (Nino-Liu et al., 2006). For this study, we chose to focus on RYMV and *Xoc* because BLS symptoms are much more specific than BLB (i.e., other bacteria can also induce BLB-like symptoms), and consequently more suitable for epidemiological studies. BLS has been reported to date in at least eight African countries (Figure 1A). It is considered as an emerging disease in some African countries, such as Burkina Faso (Wonni et al., 2011, 2014).

### Potential molecular mechanisms underlying virus-bacteria within-plant interactions

RNA silencing is a key mechanism involved in plant-virus interaction (Incarbone and Dunoyer, 2013). Viral dsRNA intermediates produced during viral replication are the inducers of anti-viral RNA silencing defense mechanism. They are processed into small interfering RNA (siRNA) duplexes through the activity of RNaseIII Dicer Like enzymes (DCL). An RNA methyltransferase, HEN1, protects siRNA duplexes from degradation by methylation and siRNA are subsequently loaded into an RNA Induced Silencing Complex (RISC) to promote specific viral RNA degradation (Incarbone and Dunoyer, 2013). Consequently, it has been demonstrated that key enzymes involved in RNA silencing pathway, such as HEN1, play a crucial role in plant defense against viruses (Boutet et al., 2003; Zhang et al., 2012). RNA silencing can spread beyond its initiation site to immunize systemic tissues ahead of viral infection. Even if signal's identity remains unclear, siRNAs are proposed to play a key role in this RNA silencing movement (Brosnan and Voinnet, 2011; Melnyk et al., 2011).

In addition to the well-established antiviral RNA silencing mechanism, recent findings point out the role of RNA silencing during plant-bacteria interactions (Pelaez and Sanchez, 2013; Seo et al., 2013). Interestingly, the rice gene encoding the methyltransferase HEN1 (*OsHen1*), involved in the stabilization of siRNA, has been shown to be the target of Transcription Activators-like Effectors (TALEs) from both *Xoo* and *Xoc* (Moscou and Bogdanove, 2009; Cernadas et al., 2014). TALEs are *Xanthomonas* proteins that are translocated into plant cells through the bacterial Type III secretion system (T3SS). They act as transcription factors by binding to promoter region and inducing expression of host plant genes to promote disease. This suggests that the manipulation of RNA silencing could be a general virulence strategy and could play an important role in plant—bacteria interactions.

### Objectives

For this research, we overall aimed to combine experimental and field work in order to document whether RYMV and *Xoc* co-exist in African rice fields, and whether they interact during simultaneous infection of the same rice plant. We detected both pathogen species in the same fields and even the same plants within rice agrosystems in Burkina Faso. In greenhouse settings, we assessed reciprocal effects of virus and bacteria on symptom expression testing several genotypes in order to test for G_P_^*^G_*P*_ interactions. In parallel, we estimated the relative effect of each pathogen on the multiplication of the other in a co-infection context and found that OsHEN1 may impact virus-bacteria within-host interactions.

## Results

### Co-occurrence of RYMV and *Xoc* reaches more than 50% of the fields in a highly infected perimeter in Burkina Faso

Estimations of RYMV and *Xoc* occurrence and co-occurrence were performed in 2015 in Burkina Faso. A total of 30 fields in three irrigated perimeters (Banzon, Karfiguela, and Douna) were surveyed (Figure 1B). The presence/absence of each disease at the studied sites are listed in Table 1. Among the 30 studied sites, both yellow mottle and BLS symptoms were observed in seven fields determined by field observation of specific symptoms (Table 1). These results were confirmed through serological (RYMV) and molecular (*Xoc*) diagnostic tests. These seven fields were located in the irrigated perimeter of Banzon, where diseases incidence was very high. Notably, BLS symptoms were found in all 12 fields in Banzon. By contrast, in Douna, yellow mottle symptoms were frequent (4 fields out of 6 visited) but BLS was not found. Both diseases were found in separate fields and at low levels in Karfiguela (Table 1).

**Table 1:** **Number of quadrats displaying specific symptoms of the ***Rice yellow mottle virus*** (RYMV), the bacterium ***Xanthomonas oryzae*** pathovar ***oryzicola*** (***Xoc***), or where the two pathogens co-occurred simultaneously, in the three investigated sites in southwestern Burkina Faso**.

**Site (number of analyzed quadrats)**	**RYMV infected fields**	***Xoc* infected fields**	**RYMV and *Xoc* co- infected fields**
Banzon (12)	7 (58.3%)	12 (100.0%)	7 (58.3%)
Douna (6)	4 (66.7%)	0 (0%)	0 (0%)
Karfiguela (12)	1 (8.3%)	2 (16.7%)	0 (0%)
Total (30)	12 (40.0%)	14 (46.7%)	7 (23.3%)

For each of the seven quadrats found to be simultaneously infected by the virus RYMV and the bacteria *Xoc* in Banzon, we analyzed a set of 16 regularly sampled plants to estimate the proportion of plants infected by either the virus or the bacteria, as well as the proportion of plants simultaneously co-infected by both pathogens. Serological diagnosis revealed that RYMV was found in 67.0% of the plants on average over seven fields, with incidence varying from 50.0 to 93.8%. We unfortunately could not distinguish the two *Xo* pathovars (see methods) and consequently applied a species-level molecular diagnostic test, revealing that that *Xo*-infected plants were 30.4% on average (with a maximum of 68.8%). Co-infected plants found to be positive for both RYMV and *Xo* were found on average at 18.8% (maximum of 37.5% in two different investigated quadrats). Detailed results obtained for each of the seven quadrats can be found in Supplementary Table S1.

### Effect of the virus on bacterial symptoms depends on the viral genotype considered

**Figure 2:**
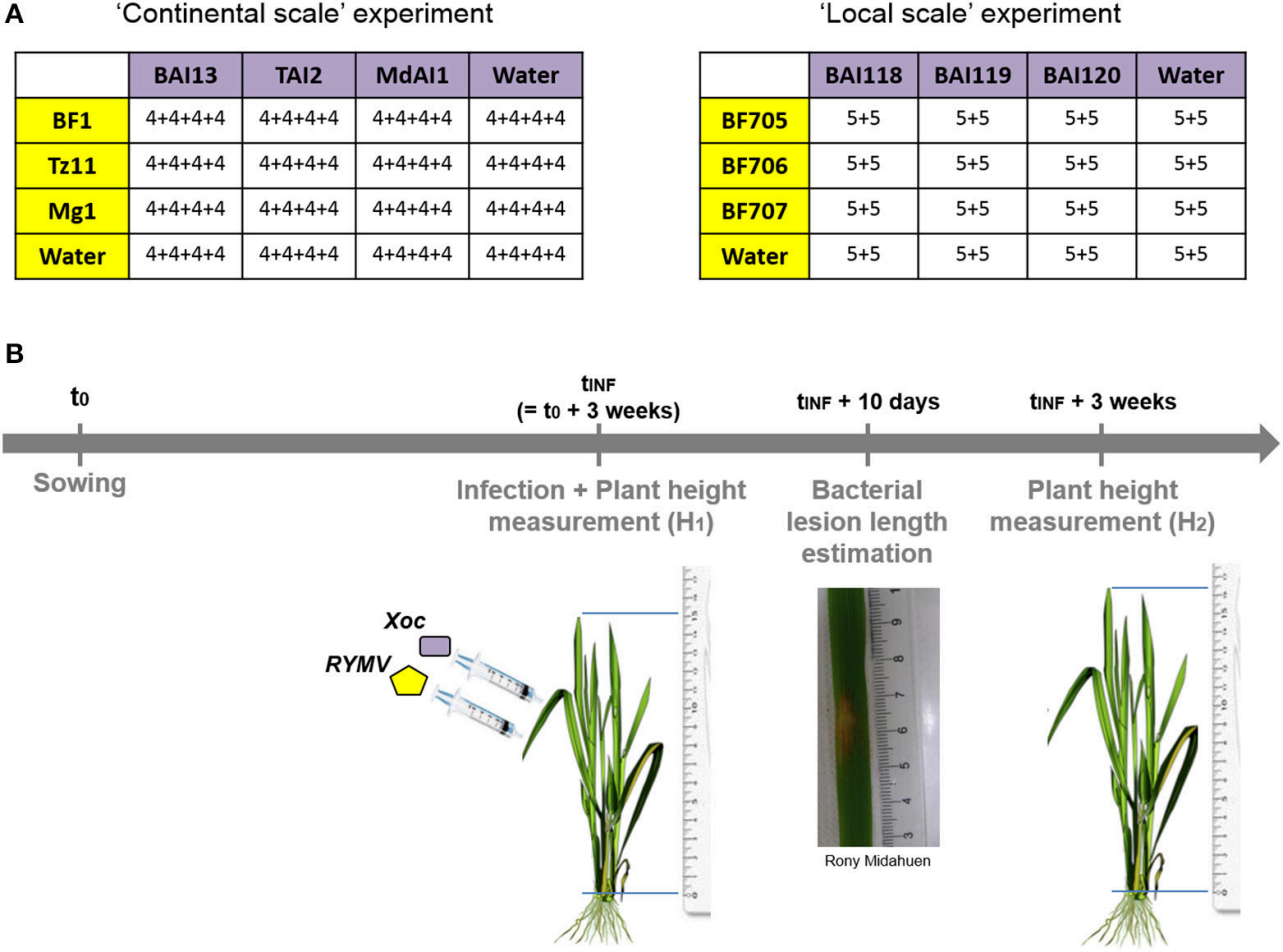
**Schematic representation of experimental design used to test for pathogen genotype by pathogen genotype interactions. (A)** The design of experimental co-infection with different viral (in yellow) and bacterial (in purple) genotypes, either at continental (Burkina Faso, Tanzania, and Madagascar) or local (within southwestern Burkina Faso) scale. Within the tables, are indicate the number of plants in each experimental block for each viral genotype by bacterial genotype combination. **(B)** The experimental set-up through time, with experimental infection by each of the two pathogens (few hours and 3–4 centimeters apart) 3 weeks after sowing, bacterial symptom estimation (through the measurement of lesion length) and plant growth estimation (from the measurement of plant height at the time of inoculation and 3 weeks after).

We aimed at testing for G_P_^*^G_*P*_ interactions for the outcome of virus-bacteria co-infection in rice in Africa. A design (Figure 2) involving two spatial scales was chosen for this purpose to get a comprehensive picture: (a) maximizing the differences between pathogen genotypes (continental scale), and (b) corresponding to the biological reality of multiple infection in the field (local scale). Viral isolates and bacterial strains were sampled within these two spatial scales. At the continental (Africa) scale, we used previously described viral isolates and bacterial strains from Burkina Faso, Tanzania and Madagascar (Figure 1A). At the local scale, we obtained RYMV isolates and *Xoc* strains from three localities in Burkina Faso (Banzon, Karfiguela, and Karankasso Sambla, Figure 1B). The viral isolates collected in Burkina Faso all belong to strain S1 (see Supplementary Figure S2), with one within the group S1ca (BF705, from Banzon) and two isolates in the group S1wa (BF707 from Karfiguela, and BF706 from Karankasso Sambla).

For each of the spatial scales, we ran an experiment under greenhouse conditions, where the three different viral isolates were co-inoculated with one of the three bacterial strains in a full factorial design. We obtained a total of 4^*^4 = 16 different treatments, including the mock inoculations. Inoculations of the virus and the bacteria were performed on the same day and the same leaf with two infiltrations a few centimeters apart using a needleless syringe. Bacterial infection with *Xoc* leads to the apparition of specific translucent lesions by 3–5 days following infiltration. We measured the lesion length at day 10 post-infiltration. RYMV specific symptoms appeared clearly 2 weeks after infection, but as they are difficult to estimate visually as a quantitative variable, we chose to use the plant growth as a proxy for observed effect of RYMV on rice (see Material and Methods).

The interaction between bacterial genotype and viral genotype was not found to be a significant explanatory variable, nor for bacterial relative symptoms, nor the relative plant growth.

**Figure 3:**
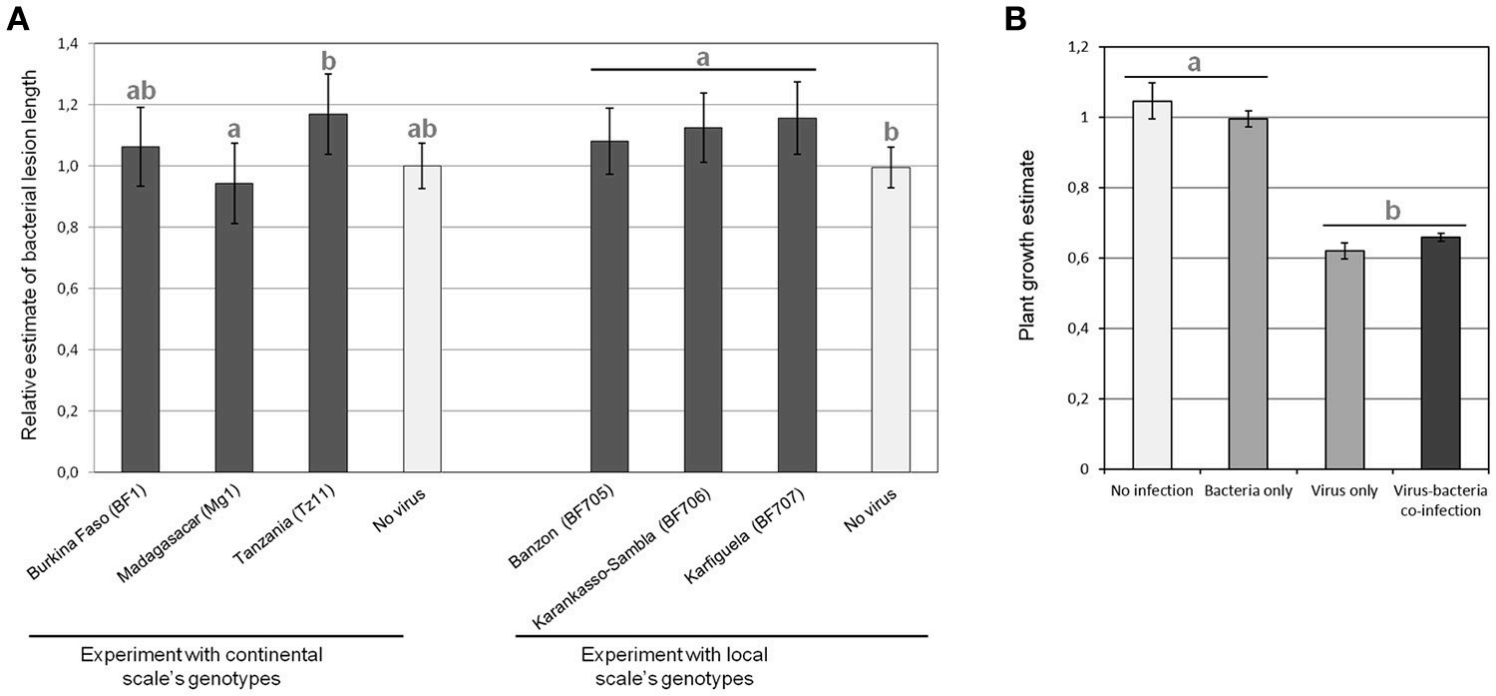
**Results in terms of observed symptoms of the experimental ***Rice yellow mottle virus*** (RYMV)-***Xanthomonas oryzae*** pv. ***oryzicola*** (Xoc) co-infections performed to test for pathogen genotype by pathogen genotype interactions. (A)** Relative bacterial lesion length, which is the length obtained in co-infection divided by the length without virus, for each considered bacterial strain, was measured after experimental infections with pathogen genotypes sampled at the continental (left) and local (right) geographical scale. Data obtained for the different bacterial strains (three for each experiment) are pooled and each bar represents the average of relative lesion length over the three bacterial strains of continental genotypes (BAI13, TAI2, and MdAI1) on the left and local genotypes (BAI118, BAI119, and BAI120) on the right. **(B)** Plant growth estimate (representing RYMV symptoms) were evaluated in experimental infections with pathogen genotypes sampled at the continental geographical scale. Each bar corresponds to the average plant growth estimates pooled for the viral isolates (BF1, Mg1, Tz11) and bacterial strains (BAI13, TAI2, and MdAI1) considered at the continental scale. Values represent the means and error bars standard deviations. Different letters indicate significant differences between groups (*p* < 0.05) when relevant.

However, for BLS relative symptoms (Figure 3A), we found a significant effect (*p* = 0.047) of the viral genotype on relative bacterial symptoms for the experiment at the continental scale. Obtained results were significantly different (*post-hoc* test, *p* = 0.036) with the viral isolate from Tanzania (Tz11) compared to the viral isolate from Madagascar (Mg1). On the other hand, at a local scale, the viral genotype had no significant effect, but the presence of the virus led to increased bacterial symptoms (*p* = 0.034).

We found that virus infection drastically reduced plant growth (Figure 3B). No effect of the particular bacterial genotype co-infecting the same rice plant could be detected on relative plant growth (representing RYMV relative symptoms). However, in the experiment at the continental scale, the presence/absence of the bacteria was marginally significant for an effect on the relative plant growth (*p* = 0.083) and we observed the following trend: when the virus was absent, the presence of the bacteria limited plant growth, while in presence of the virus, the opposite was found (Figure 3B).

### Pathogen quantifications reveal opposite reciprocal virus-bacteria interactions

**Figure 4:**
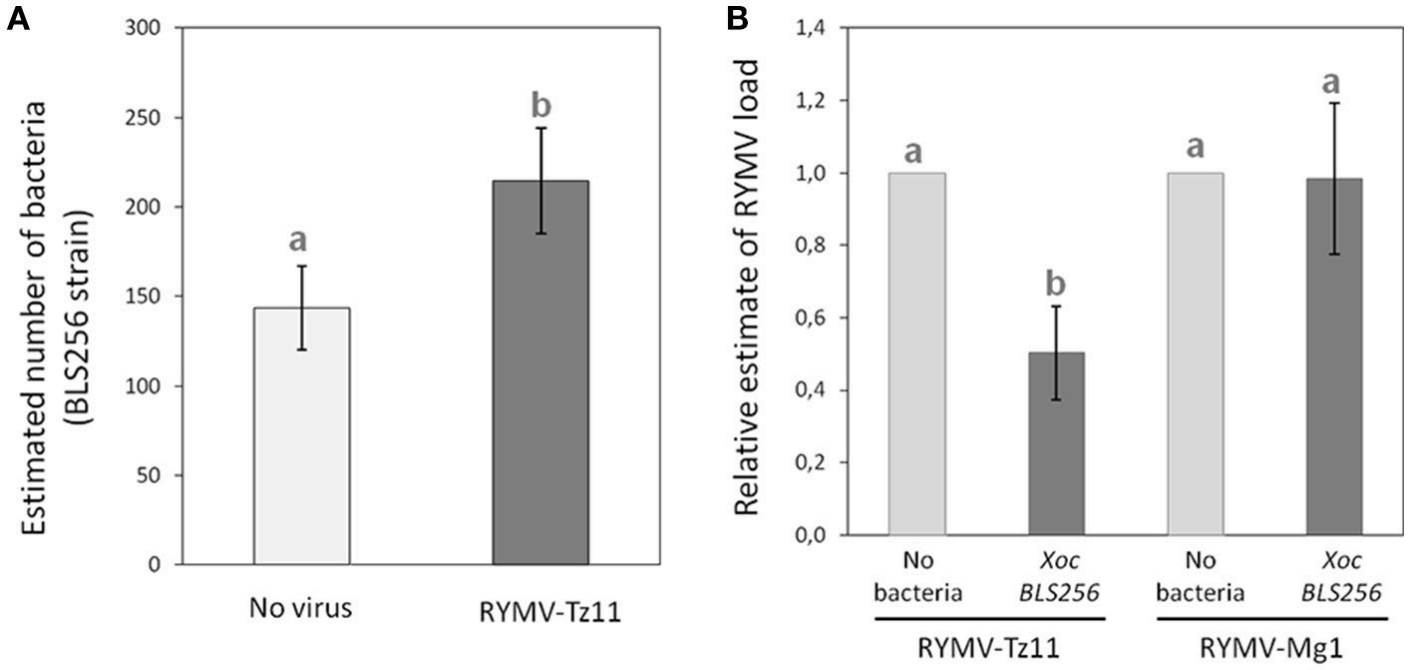
**Pathogen quantification during single-infection vs. co-infection. (A)** Bacterial load (estimated with specific qPCR) during co-infection, in dark gray, compared to single-infection in light gray. **(B)** Relative viral load (estimated using specific RT-qPCR, and normalized against the treatment with the virus only) during co-infection, in dark gray, compared to a single-infection, in light gray. Values represent the means and error bars standard deviations. Different letters indicate significant differences between groups (*p* < 0.05) when relevant.

We determined the relative viral-bacterial loads using quantitative PCR (qPCR) following experimental co-infection under greenhouse conditions for a few combinations of bacterial strains and viral isolates. We found a significant increase in bacterial load in co-infection with the virus, compared to the bacterial infection alone (*p* = 0.044; Figure 4A). When testing the effect of the presence/absence of the bacteria on virus accumulation, we found a strong effect for the viral isolate from Tanzania (Tz11, *p* = 0.012), with the virus titer being much lower (2-fold) during co-infection, compared to single viral infection (Figure 4B). No effect was observed for the viral isolate from Madagascar (Mg1, *p* = 0.932, Figure 4B).

### OsHen1 induction could underlie the effect of the bacteria on the virus

**Figure 5:**
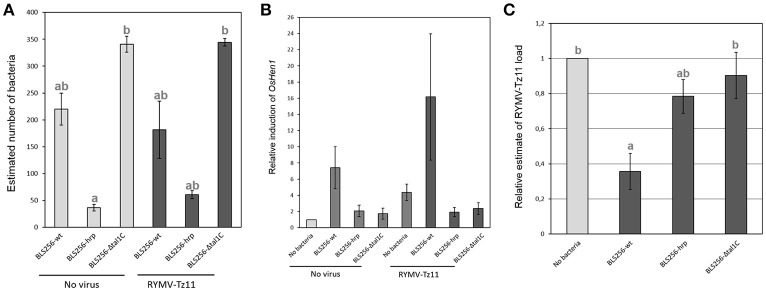
**Results obtained in the experiments testing the hypothesis of an involvement of gene silencing mechanisms in the negative effect imposed by the bacteria on the virus. (A)** Bacterial load (estimated with specific qPCR) obtained for the three bacterial relatives (BLS256 wild type and two mutants) during single-infection, in light gray, and during co-infection in dark gray. **(B)** Relative induction of the gene OsHen1 (estimated with specific q-RT-PCR) obtained for the different infection treatments. **(C)** Relative viral load (estimated with specific q-RT-PCR) obtained for the virus alone, in light gray, compared to three different co-infection treatments, in dark gray. Values represent the means and error bars standard deviations. Different letters indicate significant differences between groups (*p* < 0.05) when relevant.

Because *HEN1* is both a key regulator of anti-viral RNA silencing mechanism (Boutet et al., 2003; Blevins et al., 2006) and a target of *Xo* TALEs (Moscou and Bogdanove, 2009; Cernadas et al., 2014), we asked whether RNA silencing pathways could potentially be involved in mediating the observed negative effect of *Xoc* on the viral load of Tz11 in RYMV/*Xo* co-infection (Figure 4B). To this end, we tested the reciprocal effects of co-inoculating *Xoc* BLS256 wild-type or mutant strains with RYMV-Tz11 on pathogen accumulation (Figure 5). Philippine *Xoc* strain BLS256, a model for *Xanthomonas*-rice interactions, was chosen for molecular analysis. BLS256 carries *tal1c*, a TALE that targets the promoter of *OsHen1*, which encodes small RNA methyltransferase. Two mutant strains for BLS256 were included in the experimental design: BLS256 *tal1c* mutant M87 (BLS256 Δ*tal1c*) and BLS256 *hrp*, a Type III secretion system deficient, avirulent mutant (Cernadas et al., 2014). Tz11 viral isolate was co-inoculated onto rice leaves with either BLS256 wild-type or either mutant variant. Three days post-inoculation co-inoculated leaves were harvested and used to evaluate bacterial population by qPCR (Figure 5A) as described previously. As expected, bacterial multiplication was dramatically reduced for BLS256 *hrp* compared to the wild-type or BLS256 Δ*tal1c* (Makino et al., 2006). Notably the Δ*tal1c* mutant multiplied at higher levels than the wild type strain BLS256 (Figure 5A). A similar pattern was found when Tz11 isolate was co-inoculated with *Xoc* strains. Overall when co-inoculated using mixed inoculum, the presence of RYMV did not seem to affect bacterial multiplication as bacterial loads were similar to controls without virus (Figure 5A).

*OsHen1* mRNA accumulation was evaluated by qRT-PCR in the co-inoculated samples (Figure 5B). As expected, BLS256 strongly induces *OsHen1* mRNA accumulation whereas this induction is not observed for BLS256 variant without T3SS (*hrp*) and for the Δ*tal1c* mutant (Figure 5B). Similar relative patterns were obtained in the presence of Tz11, with a global increase in the range of *OsHen1* mRNA overaccumulation, both in presence or absence of BLS256 wild type strain. Indeed, the sample carrying Tz11 without bacteria displays a stronger *OsHen1* mRNA accumulation than the control without virus or bacteria (Figure 5B). This suggests that the virus itself is able to induce *OsHen1* mRNA overaccumulation.

To investigate a potential effect of BLS256 strains on viral replication, virus accumulation was evaluated by qRT-PCR. As RYMV moves rapidly from its inoculation site to systemic tissues (Opalka et al., 1998; Brugidou et al., 2002), this accumulation was evaluated in apical area 7 days post-inoculation (Figure 5C), and we found significant differences between the four treatments (ANOVA, *p* = 0.0155). When Tz11 isolate is co-inoculated with BLS256, virus accumulation is strongly reduced compared to the control without bacteria (*post-hoc* comparison between BLS256 and the mock without bacteria, *p* = 0.014). This effect is absent with the strain Δ*tal1c* (*post-hoc* comparison between BLS256 and Δ*tal1c, p* = 0.034), suggesting an effect of *OsHen1* induction. As this effect cannot be attributed to a difference in terms of bacterial population (Figure 5A), this suggests that Tal1c-activity may result in lower viral accumulation. T3SS-deficient BLS256-*hrp* displays an intermediary effect (Figure 5C) suggesting that among all the effectors injected through the T3SS, some would have a negative effect on viral multiplication such as *tal1C* and others would have a positive effect. The *hrp* strain is unable to inject any of these positive and negative effectors explaining its intermediate effect on virus multiplication. Because the HEN1 enzyme is directly involved in siRNA stabilization through methylation, we asked whether OsHEN1 induction by BLS256 increased viral siRNA populations in tissues infected by either the bacteria or the virus or both. Unfortunately, because of the very low amounts of viral siRNAs at this early stage post-infection (48 h), northern blot assays were not sensitive enough to allow their detection (data not shown).

## Discussion

### Virus and bacteria co-infection levels in rice agrosystems in africa

In order to face the rapidly growing demand, areas cultivated with rice have been drastically increased and practices are intensifying nowadays in West Africa (Wopereis et al., 2013). We hypothesize that such changes are likely to favor the spread of rice diseases, increasing the frequency for the fields and plants to be simultaneously infected by various pathogen species. Our results reveal that one of the studied sites, Banzon (western Burkina Faso), was highly infected by the virus RYMV and the bacteria *Xanthomonas oryzae* pv *oryzicola* (*Xoc*), and the two pathogens RYMV and *Xoc* can frequently be found together in the same field (more than half of investigated field). At the plant level, we unfortunately could not obtain such detailed data (no pathovar distinction within *Xo* species), but interestingly, two fields in Banzon presented 37.5% of plants simultaneously infected by RYMV and *Xo*. To our knowledge, this is the first study documenting jointly occurrence and incidence of various rice diseases in Africa. More detailed investigations are drastically needed to quantify infection and co-infection levels in African rice fields. Here we show that a rice disease hotspot presents high levels of virus-bacteria co-infection, and can consequently be considered as a hotspot for within-host pathogen-pathogen interactions (Louhi et al., 2015), rendering crucial to understand the potential consequences of such interactions.

### Virus-bacteria reciprocal interactions in co-infection context

Virus-bacteria interactions in plants have been less frequently studied than interactions between a couple of related species, at least within the wide groups of viruses, bacteria or fungi (Lamichhane and Venturi, 2015). However, strong effects from co-infection by unrelated species have been reported in human and animal literature (Osborne et al., 2014; Ezenwa and Jolles, 2015). The different experiments presented in this study lead to overall congruent results. In case of rice co-infection between RYMV and *Xoc*, the virus has a positive effect on bacterial multiplication (Figure 4A) and symptoms (Figure 3A), while the bacterium has a negative effect on viral multiplication (Figures 4B, 5C) and symptoms (Figure 3B).

Previous reports of virus-bacteria interactions within plants remain rare. However, negative effect of a virus on bacterial symptoms was described in gourd plants (Shapiro et al., 2013). The authors showed that *Zucchini yellow mosaic virus* (ZYMV)-infected plants exhibit delayed wild symptoms due to *Erwinia tracheiphila* infection. More generally, the outcome of pathogen-pathogen interactions can be either positive or negative (and not necessarily the same for the two pathogens in co-infection, as illustrated by our study) and remains unpredictable (Tollenaere et al., 2016).

### Pathogen genotype by pathogen genotype interactions and evolutionary consequences

Investigating pathogen genotype by pathogen genotype (G_P_^*^G_P_) interactions for co-infection outcome is an important research issue because of the consequences of such interactions for the maintenance of pathogen population genetic diversity (Seppala et al., 2009). In addition to Seppala's pioneer studies (Seppala et al., 2009, 2012) documenting the effect of different pathogen genotypes on co-infection issue in terms of infection success, a few studies very recently investigated this issue in terms of overall virulence, or host survival (Bose and Schulte, 2014; Louhi et al., 2015).

Here, we could not detect an effect of G_P_^*^G_P_ interactions on relative bacterial symptoms or relative plant growth (used as a proxy for RYMV symptoms in our study). This may be due to the methods used to estimate symptoms that remain imprecise, increasing variability in the dataset. The possibility to estimate pathogen multiplication in this experiment could have made it possible to evidence G_P_^*^G_*P*_ interactions.

However, we could show that the specific viral isolate can affect the outcome of co-infection with a positive effect on bacterial symptoms only observed for some viral isolates, in particular the Tanzanian isolate Tz11 (congruent results in the different experiments, see Figures 3A, 4B). Similarly, no ${\text{G}}_{\text{P}}^{*}$G_P_ interactions have been observed for the effect of co-infecting bacterial strains on nematode survival (Bose and Schulte, 2014), nevertheless, the interaction mechanism depends on the particular bacterial strain considered. As in our study, the molecular mechanisms involved would consequently depend on the particular pathogen genotype considered.

### Insight into molecular mechanisms

Molecular mechanisms underlying interactions between co-infecting pathogens remain poorly documented. However, an involvement of RNA silencing mechanisms has been evidenced in a few cases, among which the well-documented synergism between Potato virus X (PVX) and Potato virus Y (PVY) (Rochow and Ross, 1955). In *Nicotiana tabacum*, PVX infection leads to the expression of VSR HcPro responsible for PVX hyper accumulation (Gonzalez-Jara et al., 2005). Two examples illustrate how RNA silencing mechanisms may be involved in virus-bacteria within-host interaction in Arabidopsis: *Pseudomonas* bacteria growth is increased in presence of the *Turnip Mosaic Virus* (Navarro et al., 2008) and the *Cauliflower mosaic virus* (CaMV) (Zvereva et al., 2016). In both cases, viral proteins (HcPro and P6 respectively) suppress the plant's first line of defense (PAMP Triggered Immunity, PTI), facilitating the multiplication of bacteria (Navarro et al., 2008; Zvereva et al., 2016).

Here we show that *OsHen1* mRNA accumulation induced by Tal1C from BLS256 results in a decrease in viral accumulation in systemic tissues (Figure 5C). It has been previously demonstrated that *Arabidopsis hen1* mutant displays a hypersensitive response to *Turnip crinkle virus* (TCV) and to *Cucumber mosaic virus* (CMV) leading to increased viral multiplication (Boutet et al., 2003; Zhang et al., 2012). This demonstrated the crucial role of HEN1 in anti-viral RNA silencing defense mechanism. Consequently, it is conceivable that the induction of *Hen1* mRNA accumulation has the opposite effect of decreasing viral multiplication. As HEN1 is involved in protecting siRNA from degradation (Li et al., 2005), overaccumulation of HEN1 could lead to siRNA overaccumulation. In our case, for Tz11/BLS256 co-inoculation, we can predict that viral siRNA produced by the host RNA silencing defense mechanisms accumulate more in the presence of BLS256 because of *tal1*C mediated *OsHen1* mRNA induction. This hypothesis needs to be verified by a more sensitive technique than northern blot used here such as next-generation sequencing technology. siRNAs are involved in the spread of RNA silencing signal in systemic tissues (Brosnan and Voinnet, 2011; Melnyk et al., 2011). Therefore, it is likely that the anti-viral RNA silencing signal would spread more efficiently in the case of Tz11/BLS256 co-inoculation than in the case of a single viral inoculation. This enhanced viral immunization of systemic tissues ahead of viral infection would explain the reduction in Tz11 accumulation observed in the case of Tz11/BLS256 co-inoculation (Figure 5C). Our results suggest that the efficiency of the RNA silencing response as a defense mechanism against pathogens would be dependent on the populations of pathogens co-infecting the host, as illustrated here where the Mg1 and Tz11 isolates have different effects in co-infection with *Xoc*. Here again, RNA silencing could play a role in this pathogen genotype specificity. Indeed, it has been demonstrated that RYMV encodes a protein acting as a suppressor of RNA silencing with variable activity depending on the viral isolate of origin: Mg1 carries a weak RNA silencing suppressor whereas Tz11's displays a strong activity (Sire et al., 2008; Lacombe et al., 2010). A more detailed molecular characterization of this interaction is needed to validate the direct or indirect involvement of RYMV RNA silencing suppressor in the RYMV-*Xoc* co-infection scenario.

### Perspectives

This work highlights the relevance of studying virus-bacteria multiple infection in rice in Africa, encouraging further research on co-infection in plants (Tollenaere et al., 2016), particularly considering distantly related pathogen pairs. Indeed, ecologically-relevant interactions are not restricted to phylogenetically related taxa (Spoel et al., 2007; Tack et al., 2012). Further understanding of the causes of the diversity of infection outcome in co-infection and the dynamics of multi-pathogen assemblage relies on a more exhaustive description of microbial communities and the consideration of the true diversity of pathogens associated with any given host (Vayssier-Taussat et al., 2014). The recent raise of next-generation sequencing technologies (Knief, 2014) opens up exciting possibilities to document the tremendous diversity of microbes associated with a single plant (e.g., the phytobiome), many of which may be pathogenic. In this context, integrating the pathogen into its whole biotic environment (e.g., the pathobiome concept) has the potential to expand our understanding of infection, epidemiology and evolution of pathogen populations (Vayssier-Taussat et al., 2014). This would be a requisite for the investigation of potential interactions between pathogens and any microbes inhabiting plants (Seabloom et al., 2015) and the application of the pathobiome concept for plant pathogens (Vayssier-Taussat et al., 2014).

## Materials and methods

### Field estimation of co-infection levels in Southwestern Burkina Faso

#### Study sites

In 2015, we aimed at estimating RYMV-BLS co-infection levels in the following irrigated perimeters (Figure 1B): Banzon (GPS: N 11.31955; 04.80978), Karfiguela (GPS: N 10.68347; W 04.81605), and Douna (GPS: N 10.62733; W 05.10107). In these three sites, we randomly selected from aerial pictures a set of 20^*^20 meters quadrats, that we investigated and sampled. In Banzon and Karfiguela, where co-infection had been reported in 2014 (see below), we visited 12 quadrats, while 6 were studied in Douna, resulting in a total of 30 investigated fields across the three sites. In every case, we obtained permission from the farmers to work (symptom observations and leaves sampling) in their fields.

#### Field procedure

Each selected quadrat was carefully inspected for RYMV and/or BLS symptoms. To confirm the presence/absence of each disease within each investigated quadrat, we sampled symptomatic leaves. Yellow mottle symptomatic leaves were kept dry in a plastic bag containing silica gel for further serological diagnosis. Bacterial streak symptomatic leaves were kept cool and were frozen when back at the laboratory before the molecular detection procedure.

Additionally, we sampled 16 plants regularly, on a 4^*^4 grid covering the quadrat and materialized using wooden stakes. For each of the 16 plants, we sampled three leaves (including symptomatic leaves if observed) and kept them dry using a plastic bag containing silica gel.

#### Molecular diagnostic

In the fields where yellow mottle symptoms were observed, we ran specific serological diagnostic test (ELISA) (Ndjiondjop et al., 1999; N'Guessan et al., 2000; Traoré et al., 2008), on sampled symptomatic leaves. Approximately three centimeters of dried leaves were homogenized in 1-mL sterile water in extraction bags (Bioreba, Reinach, Switzerland). We followed the standard procedure of anti-RYMV DAS-ELISA (Double Antibody Sandwich Enzyme Linked Immuno Assay) test with the RYMV-Mg antiserum described in Ndjiondjop et al. (1999) and N'Guessan et al. (2000). Absorbance at 405 nm was read using a spectrophotometer (Metertech 960) and the detection threshold was set following Traoré et al. (2008).

For the bacterial streak symptomatic leaves collected and kept frozen, we also used approximately three centimeters of leaves, homogenized in 1-mL sterile water in Bioreba extraction bags. One microliter of obtained solution was used directly for *Xanthomonas*-specific multiplex PCR (Lang et al., 2010), with 0.4 μL of each of the six primers (Xo3756F, Xo3756R, Xoo281-80F, Xoo281-80R, Xoc3866F, and Xoc3866R) at 5 μM and 4 μL of 5x HOT FIREPOL Blend Multiplex (Solis BioDyne, Tartu, Estonia) in a final volume of 16 μL. Initial denaturation of 15 min at 95°C was followed by 35 cycles (30 s at 95°C, 30 s at 60°C, 2 min at 72°C) and a final extension of 10 min at 72°C. PCR products were run on 1.5% agarose gels to check the presence/absence of *Xo, Xoo*, or *Xoc* specific bands.

In the fields where both RYMV and *Xoc* had been reported, we analyzed the 16 collected plants in order to estimate plant-level co-infection levels. To this end, the dried leaf samples were submitted independently to both RYMV serological diagnosis test and *Xo*-specific multiplex PCR following the methodology described above. Unfortunately, the resolution of agarose gel performed did not allow to distinguish between *Xo, Xoo*, or *Xoc* specific bands and we could only consider the presence/absence of *Xanthomonas oryzae* (not the two different pathovars) within in the RYMV-*Xoc* co-infected fields.

### Experimental assessment of G_P_^*^G_*P*_ interactions for symptom development

The experimental design used for this section is presented in Figure 2.

#### Viral isolates and bacterial strains used

Two spatial scales were considered for the sampling and selection of viral isolates and bacterial strains. At the continental scale (Africa), we used viral isolates and bacterial strains collected in previous studies and originating from three countries: (1) Burkina Faso BF1 (Pinel et al., 2000) and BAI13 (Wonni et al., 2014), (2) Tanzania Tz11 (Fargette et al., 2004) and TAI2 (Poulin and Szurek, unpublished), and (3) Madagascar Mg1 (Pinel et al., 2000) and MdAI1 (Poulin et al., 2014), for RYMV and *Xoc* respectively.

At the local scale, we visited rice fields on October 5-6 2014 in Southwestern Burkina Faso. Both RYMV and BLS symptoms were found in the same fields in three localities: irrigated perimeters of Banzon (GPS: N 11.31955; 04.80978) and Karfiguela (GPS: N 10.68347; W 04.81605), and in a rainfed farmer's field in Karankasso Sambla (GPS: N 11.24732; W 04.56256). Viral isolates obtained were named respectively: BF705 from Banzon, BF706 from Karankasso Sambla and finally BF707 from Karfiguela.

Virus-infected rice leaves have been collected and dried in plastic bags containing silica gel. RYMV-specific ELISA diagnostic test was performed as previously described (Traoré et al., 2008) and confirmed the visual diagnostic of RYMV. Genetic characterization of collected samples was performed though sequencing of the CP region as described previously (Pinel et al., 2000). Briefly, the samples were first submitted to RNA extraction (using RNeasy Mini Kit, Qiagen), reverse transcription (using the following primer RYMV II: CTCCCCCACCCATCCCGAGAATT), amplification (using RYMV II and RYMV III: CAAAGATGGCCAGGAA) and sequencing of CP region. The CP sequences from the 3 isolates tested in this study and a set of 300 CP sequences from 17 African countries representing the RYMV genetic diversity (Pinel-Galzi et al., 2009) were aligned using CLUSTAL X with default parameters (Thompson et al., 1994). A model selection procedure accessed through MEGA6 (Tamura et al., 2013) was run to select the best fitted model of nucleotide substitution. The Kimura-2 model with a rate variation and an invariant rate class (K2+G+I) provided the best fit. The maximum-likelihood phylogenetic tree was inferred using MEGA6.

BLS-infected rice leaves were sampled and kept in a cold box until freezing. Bacterial isolation was performed at 28°C on PSA medium with antibiotics (actidione 50 mg/L; cephalexin 40 mg/L; kasugamycin 20 mg/L). Isolated bacterial strains obtained were preserved in PSA-glycerol medium for long-term storage at −80°C. They were named BAI118 (from Banzon), BAI119 (from Karfiguela) and BAI120 (from Karankasso Sambla).

#### Experimental conditions and inoculums set-up

All experiments described in this study were performed under greenhouse conditions (IRD Montpellier, France) with cycles of 12 h of light at 28°C and 80% relative humidity (RH) and 12 h of dark at 25°C and 70% RH. We used rice seeds of the variety IR64 known to be highly susceptible to both RYMV and *Xoc*.

The first inoculation was always performed 3 weeks after sowing rice seeds. The preparation of viral inoculum involved crushing 1 g of RYMV-infected leaves using mortar and pestle. We added 20 mL of 0.1 mM phosphate buffer (1 mM KH_2_PO_4_ and 1 mM Na_2_HPO_4_, pH 7.2) and this solution was centrifugated at 4,000 rpm during 2 min. The supernatant was used as viral inoculum. For bacterial inoculum, we cultured *Xoc* on PSA medium (10 g peptone, 10 g sucrose, 1 g glutamic acid, 16 g agar, in 1 L of H_2_O) for 48 h. A bacterial suspension from the fresh plate was diluted in sterile water to obtain a DO_600_ = 0.5 inoculum. Both viral and bacterial inoculations were performed by infiltrating inoculum solution using a needleless syringe.

#### Experimental procedures

Full-factorial design was used for each of the two spatial scales. Three viral genotypes and three bacterial genotypes were considered at each spatial scale, with all possible combinations tested, including the single infections of each genotype set as controls. This design resulted in 4^*^4 = 16 treatments for each experiment.

Experiments at continental scale were performed in 4 blocks (inoculation dates: 15/12/2014; 09/01/2015; 06/02/2015; and 13/03/2015), while experiments at local scale were performed in 2 blocks (inoculation dates: 30/04/2015 and 29/05/2015). Each experimental unit corresponds to a 1-L pot containing three IR64 (a highly susceptible variety) rice plants.

Both viral and bacterial inoculations were performed on the same day, 3 weeks after sowing. The preparation of viral inoculums was performed by crushing 1 g of RYMV-infected leaves using mortar and pestle and adding 20 ml of water. This solution was centrifugated at 4000 rpm during two minutes and we kept the supernatant as viral inoculum. For bacterial inoculum, bacteria were grown on PSA medium (see above) and diluted in water to obtained a concentration of OD = 0.5. Infiltrations were done using sterile needleless syringes on the same leaf: firstly, the virus, and then, the bacteria, approximately at 3 centimeters closer to the stem.

Infected plants were monitored during 4 weeks post-inoculations. BLS specific symptoms generally appear 3–4 days post-inoculation. They were estimated by measuring the lesion length, 10 days after inoculation. Viral infection leads to the appearance of yellow mottle symptoms on newly developed leaves, 10 days post-infiltration at the earliest. The whole plant then displays symptoms with leaves turning orange and drying. Disease caused by RYMV can ultimately result in plant death in susceptible varieties. Such RYMV specific symptoms are difficult to estimate as a quantitative variable. We chose therefore to use plant growth as a proxy for virus effect on the plant as RYMV is known to drastically affect the growth of rice plants (Ghesquiere et al., 1997). We consequently measured plant height firstly at the time of inoculation (H_1_) and at the end of the experiment, 3 weeks post-inoculation (H_2_) and calculated plant growth as (H_2_-H_1_)/H_1_.

#### Statistical analyses

For each co-infected plant, we estimated the relative BLS symptom length during co-infection, by dividing the observed lesion length in co-infection with RYMV by the average lesion length in single infection with the given *Xoc* strain for each experimental block.

Similarly, we evaluated relative plant growth (estimate for relative RYMV symptoms) by dividing the observed plant growth in co-infection with *Xoc* by the average plant growth in single infection with the considered RYMV isolate for each experimental block.

For each of these two relative symptoms estimates, results were analyzed using Generalized Linear Models in R (R Core Team, 2014). We first tested whether the bacterial genotype by viral genotype interaction had a significant effect; if not, we used the co-infecting pathogen genotype or finally the presence/absence of co-infecting pathogen as explanatory variable.

All the figures showing the results were designed using Microsoft® Office Excel, with error bars representing standard deviations.

### Pathogen quantification in co-infection vs. single infection context

To assess whether the differences in symptoms would translate into differences in terms of pathogen accumulation, we performed experimental infections of the highly susceptible variety IR64, and quantified viral and bacterial loads using specific quantitative PCR assays. Such experiments were performed using (1) the RYMV isolate Tz11 or Mg1, which were chosen because of their contrasted efficiency in suppressing RNA silencing, with strong and mild suppression for Tz11 and Mg1 respectively (Sire et al., 2008); and (2) the bacterial strain BLS256, originating from the Philippines and a model for *Xoc*.

#### Effect of the virus on bacterial load

Bacterial inoculation consisted of 10 spots of infiltration. For the experiment involving simultaneous co-infection, the virus was inoculated either jointly with the bacteria (same syringe) while control “single-infection” treatment contained only bacteria. On the other hand, for the experiment involving delayed co-infection, we inoculated the virus first, and then 10 spots of bacteria were infiltrated on a distinct leaf. The delay between viral and bacterial inoculations was either very short (both inoculations performed in a row, less than 30 min apart) or set to 7 days. We obtained two biological replicates for each experiment. The area around these 10 spots (8 cm leaf fragment) was sampled 48 h after infiltration. Each biological sample is a pool of two inoculated leaves. Samples were placed in liquid nitrogen and then stored at −80°C prior to DNA extraction, using the DNeasy Qiagen kit (Qiagen, Hilden, Germany).

Quantification of bacterial load was performed through a new qPCR procedure designed for the purpose of this study. This method is based on a *Xo*-specific primer pair (Xo3756F and Xo3756R) widely used for *Xo* molecular diagnostic (Lang et al., 2010). We used the qPCR product as a method to quantify the bacteria. Bacterial quantification method was assessed by comparing the results obtained with classical bacterial colony counting, and this part is described in the Supplementary Information S3.

#### Effect of the bacteria on viral titer

We used mixed inoculums of virus and bacteria and infiltrated the inoculums with 10 contiguous spots on the same leaf. This experiment was repeated five times. Apical zones were sampled by dissecting the plant with a scalpel, 7 days after inoculation. Each sample consisted in a pool of two apical zones sampled. RNA was extracted using the Tri Reagent (Sigma® Aldrich, St Louis), following the manufacturer's recommendations. Obtained RNAs were diluted in RNA-free water. Their concentration was estimated using Nanodrop for λ = 230 nm (Thermo Scientific, Wilmington, USA), and quality was assessed by migrating on 1% agarose gels. Viral load was estimated using RYMV-specific qRT-PCR assay (Poulicard et al., 2010).

#### Data analysis

The results obtained for single infection were compared to the corresponding co-infection treatments using Student's *t*-test in R software. For the effect of the virus on bacterial load, we pooled the three experiments performed despite small differences in infection methodology (e.g., simultaneous or delayed co-infection) so as to attain a sufficient sample size to compare bacterial load in presence or absence of the virus.

### Molecular mechanisms of virus-bacteria interactions: testing of the involvement of OsHen1 on the effect of the bacteria on the virus

Based on the crucial role RNA silencing mechanisms play in plant defense against both viruses and bacteria, we hypothesized such mechanisms could help explain the within-plant virus-bacteria interactions. Interestingly, the rice gene encoding HEN1 (*OsHen1*), is known to be both (1) involved in the stabilization of siRNA by methylation and (2) the target of *Xoo* and *Xoc* effectors (Perez-Quintero et al., 2013; Cernadas et al., 2014). We therefore speculated that the negative effect the bacteria *Xoc* has on the virus RYMV could be mediated by *OsHen1* expression.

#### Mutant strains used

We used the previously published wild-type BLS256, the mutant strain BLS256 *hrp*, a Type III secretion system deficient, avirulent mutant and the mutant strain M87 with a single disruptive insertion mapping to tal1c (kanamycin resistant), labeled BLS256 Δ*tal1c* (Cernadas et al., 2014).

#### Experimental procedures

Experimental infections were performed on IR64 rice plants, following the same methodology as described above for the effect of the bacteria on the virus (see Effect of the Bacteria on Viral Titer). Inoculated leaves were sampled 48 h post-inoculation and were used for bacterial load quantification of bacterial load using *Xo*-specific qPCR procedure (see Effect of the Virus on Bacterial Load). In addition, *OsHen1* expression was quantified through quantitative reverse transcriptase PCR. Seven days after inoculation, apical zones were sampled and RYMV load was assessed following the same methodology as described above (see Effect of the Bacteria on Viral Titer). The experiment was replicated three times.

RNA was extracted with Trizol and treated with RNase-free DNase I following manufacturer's recommendations. 200 ng RNA was reverse transcribed with Invitrogen Superscript III kit using oligo-dT primers following manufacturer's protocol. Gene expression was monitored using SYBR-Green (MESA Blue). Two microliter diluted cDNA template (1 template/10 water) was added to 5 μL master mix comprising of 3.5 μL MESA Blue, 0.35 μL forward primer (10 μM), 0.35 μL forward primer (10 μM) and 0.8 μL H_2_O for a 7 μL total volume. qPCR was performed on Stratagene MX3005P (Stratagene, La Jolla, CA), using 384-well plates, and with the following cycles: 10 min activation at 95°C, 40 cycles of 95°C for 15 s 60°C for 20 s and 72°C for 40 s. Differential expression was calculated comparing treatment vs. water control using the ΔCt method (Vandesompele et al., 2002). cDNA quantities were normalized using elongation factor-1 alpha gene (sequence primers: GAAGTCTCATCCTACCTGAAGAAG and GTCAAGAGCCTCAAGCAAGG) as a reference gene.

#### Data analysis

Results obtained in the three different experiments were pooled for statistical analyses. The effect of the different bacterial strains on estimated parameters was assessed using Anova, followed by Tukey *post-hoc* test in case of significant result, in R software.

## Author contributions

CT supervised the whole research, along with SL, SC, and CB for the aspects of molecular mechanisms of interactions. IW and SC brought their bacteriologist's expertise, while EH, DS, SL, and CB provided their knowledge in virology. The experiments on molecular mechanisms were conceived and performed by SL, CN, SC, and JJ. The design of the field work protocol, as well as field observations and sampling were performed by IW, DS, MB, and CT. Molecular and serological diagnostic were done by FG and MB. Co-infection experiments at continental and local scale were performed by CT, following advice from EH. Molecular characterization of new RYMV isolates was performed by EH. Finally, CT, SL, and CB wrote a first draft of the article, which was improved by suggestions from all authors.

## Funding

This study was facilitated by the formal partnership between IRD and INERA within the international lab “LMI Patho-Bios: Monitoring Plant Pathogens in West Africa.” This project is supported by the French Agropolis Foundation (Labex Agro-Montpellier: ANR-10-LABX-0001-01), with two projects (reference IDs 1403-041 and 1504-004).

### Conflict of interest statement

The authors declare that the research was conducted in the absence of any commercial or financial relationships that could be construed as a potential conflict of interest.
