# Supplementary material for: Virus-Bacteria Rice Co-Infection in Africa: Field Estimation, Reciprocal Effects, Molecular Mechanisms, and Evolutionary Implications
Source: Front Plant Sci. 2017 May 1;8:645. doi: 10.3389/fpls.2017.00645 (PMC5410622; doi:10.3389/fpls.2017.00645)
Supplement: Supplementary file 1 [file DataSheet1.docx]

**SUPPLEMENTARY MATERIAL**

**Supplementary Table S1**

Proportion of plants infected by the virus *Rice yellow mottle virus* (RYMV), by the bacteria *Xanthomonas oryzae* (*Xo*) and co-infected by both pathogens in seven quadrats found to be simultaneously affected by RYMV and *Xo* pathovar *oryzicola* in the irrigated perimeter of Banzon (western Burkina Faso).

Incidence and co-incidence values are shown as percentages, estimated from diagnosis tests performed on 16 regularly sampled plants within each quadrat. Incidence of “<6.2” is indicated in the fields where all the 16 sampled plants revealed negative.

| Quadrat | | Incidence RYMV  (%) | Incidence *Xo*  (%) | Co-incidence RYMV-*Xo* (%) |
| --- | --- | --- | --- | --- |
| 1 | BZ02 | 68.75 | 62.5 | 37.5 |
| 2 | BZ04 | 75 | < 6.2 | < 6.2 |
| 3 | BZ06 | 50 | 68.75 | 37.5 |
| 4 | BZ07 | 75 | 37.5 | 25 |
| 5 | BZ09 | 93.75 | 25 | 25 |
| 6 | BZ10 | 56.25 | 18.75 | 6.25 |
| 7 | BZ11 | 50 | < 6.2 | < 6.2 |

**Supplementary Figure S2**

Phylogenetic relationships of West African isolates. The phylogenetic tree was reconstructed from 303 coat protein gene sequences including a corpus of representative isolates from 17 countries (Pinel-Galzi et al., 2009) and the newly described isolates from Burkina Faso (BF705 from Banzon, BF706 from Karankasso Sambla and BF707 from Karfiguela) in bold. The East African strains were represented as compressed branchs.

**
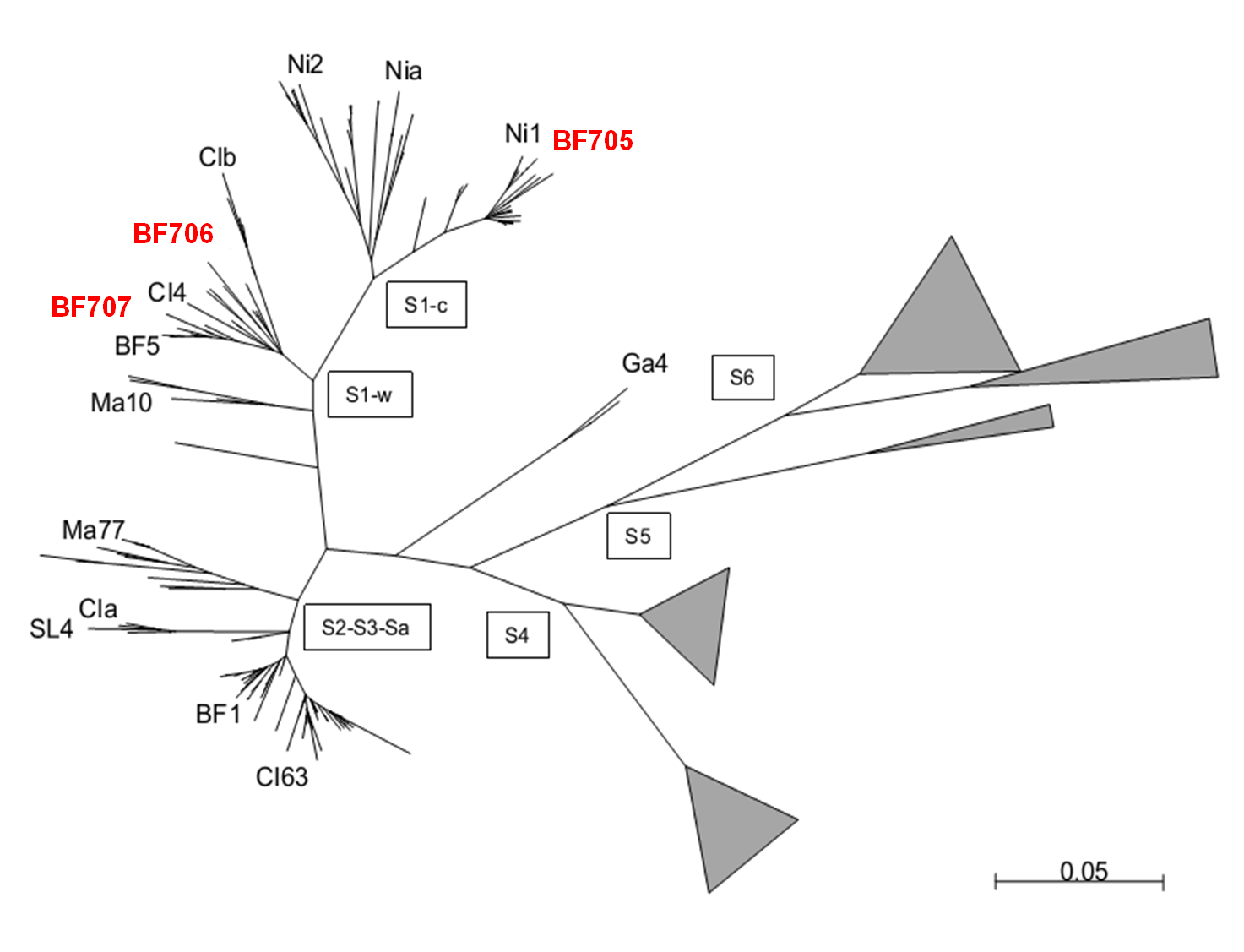
**

**Supplementary Information S3**

Assessement of a *Xanthomonas oryzae* -specific quantitative PCR procedure designed to estimate bacterial load in infected rice leaves

In order to assess the new technique, we compared the resultas obtained using qPCR method with the classical colony counting methodology. To this purpose, we prepared 26 samples by inoculating three-weeks IR64 rice under controlled conditions, with one of of seven different bacterial strains of *Xanthomonas oryzae* pv. *oryzae* and *Xanthomonas oryzae* pv. *oryzicola*. Infiltrations were performed with bacterial suspension at DO_600_ = 0.5. Half of the samples consisted of one leaf with only one infiltration spot while the second half constitued of four contiguous infiltration spots in the same leaf. A 4 cm long leaf segment comprising infiltration spot(s) was collected 72 hours post-infiltration, and ground into a fine powder using the Qiagen TissueLyser system. Ground material was suspended in 1mL sterile water, this volume being divided to be used for qPCR on the one hand and for colony counting on the other hand.

DNA extraction was performed using the DNeasy Plant Mini Kit (Qiagen Valencia, CA) following manufacturer’s instructions. The proposed qPCR protocol is based on a *Xo*-specific primer pair routinely used for Xo diagnostic: Xo3756F and Xo3756R (Lang et al., 2010). The qPCR mix was prepared following the manufacture’s protocol: 12.5µl of 2X Brilliant II SYBR Green QPCR Master Mix (Agilent Technologies, Santa Clara, CA), 0.5µl of each primer and 5 of DNA sample in a final volume of 25µl.

In parallel, we estimated the bacterial concentration using stabdard colony counting method (see for example ). Briefly, 5µl drops of a dilution series were spotted as triplicates onto selective PSA plates containing cycloheximide (50mg/L), kasugamycin (20mg/L) and cephalexin (40mg/L).

The relationship between the logarithm of bacterial counting (logC) and the cycle threshold (Ct) was assessed using the function “cor.test” in R software (Team, 2014). We obtained a strong correlation (rho = -0.898 and *p* = 4.9.10^-^10) between the results obtained by classical colony counting (logC) and the results obtained through the newly described quantitative PCR method (Ct).
